# Effects of Seawater Acidification on Cell Cycle Control Mechanisms in *Strongylocentrotus purpuratus* Embryos

**DOI:** 10.1371/journal.pone.0034068

**Published:** 2012-03-27

**Authors:** Sean P. Place, Bryan W. Smith

**Affiliations:** Department of Biological Sciences and The Environment and Sustainability Program, University of South Carolina, Columbia, South Carolina, United States of America; Institute of Marine Research, Norway

## Abstract

Previous studies have shown fertilization and development of marine species can be significantly inhibited when the pH of sea water is artificially lowered. Little mechanistic understanding of these effects exists to date, but previous work has linked developmental inhibition to reduced cleavage rates in embryos. To explore this further, we tested whether common cell cycle checkpoints were involved using three cellular biomarkers of cell cycle progression: (1) the onset of DNA synthesis, (2) production of a mitotic regulator, cyclin B, and (3) formation of the mitotic spindle. We grew embryos of the purple sea urchin, *Strongylocentrotus purpuratus*, in seawater artifically buffered to a pH of ∼7.0, 7.5, and 8.0 by CO_2_ infusion. Our results suggest the reduced rates of mitotic cleavage are likely unrelated to common cell cycle checkpoints. We found no significant differences in the three biomarkers assessed between pH treatments, indicating the embryos progress through the G_1_/S, G_2_/M and metaphase/anaphase transitions at relatively similar rates. These data suggest low pH environments may not impact developmental programs directly, but may act through secondary mechanisms such as cellular energetics.

## Introduction

Artificial changes in intracellular and extracellular pH have long been known to impact sea urchin sperm motility, embryogenesis, and developmental patterns [1], [2]. In recent years, a growing body of literature has highlighted the significant impacts anthropogenically driven decreases in sea surface pH can have on marine life, and in particular, marine calcifiers [3], [4]. While marine embryos are generally considered to be equipped with robust defense mechanisms against predictable environmental changes [5], exposure to near –future high CO_2_, low pH environments may prove to be a particular challenge to early life-history stages of marine larvae. Previous studies investigating the impact of ocean acidification on echinoderms have demonstrated a wide range of effects, including reduced fertilization and larval development rates, survival rates and body size [4], [6]. While this research has highlighted many developmental and morphological impacts on marine calcifiers, we still have little insight into the cellular mechanisms involved, and thus our ability to understand the potential for adaptation among organisms with long generation times remains largely dependent on measurements of genetic variation and demographic parameters [7].

Recent studies performed at the level of the gene transcript in sea urchin larvae are beginning to shed light on the molecular underpinnings of the biological response to low pH environments. These studies highlight a general decrease in a broad range of biological processes for developing larvae suggesting the mechanism may be related to regulation of gene expression [8]–[11]. However, low pH seawater has been reported to hinder the cleavage of developing embryos even though a vast majority of mRNA sequences found in embryo polysomes are still maternally derived [12], suggesting low pH environments can impact multiple levels of biological regulation. To gain insight into some of the earliest cellular pathways impacted during development in a high CO_2_ world, we can look to early developmental studies for formulation of plausible hypothesis.

Upon sperm binding to the egg, several physiological changes occur within the egg in a manner that is critical for activation of the developmental program. Most notable is a rapid increase in intracellular Ca^2+^, which has been documented in eggs of every species studied to date [13]–[16]. Shortly after the calcium ion levels rise in a sea urchin egg, its intracellular pH also increases. This rise in intracellular pH is achieved through the exchange of sodium ions from the seawater and hydrogen ions from the egg, and it is thought that the pH increase and the calcium ion elevation act together to stimulate new protein synthesis and DNA synthesis [17]–[24]. Thus, we hypothesize that low pH seawater retards development by overwhelming the ion gradient established by the exchange of internal H^+^ ions with external Na^+^ ions, essentially blocking the onset of DNA synthesis in developing embryos.

Alternatively, a low pH environment may simply be cytotoxic, or more specifically, genotoxic to a developing embryo. The ability of cells to maintain genomic integrity is essential for cell survival and proliferation, and as such, organisms have developed a robust system to maintain DNA stability during mitosis referred to as cell cycle checkpoints. These common regulatory mechanisms control key transitions in the cell cycle primarily through the cyclical activation and deactivation of protein kinase complexes composed of cyclins and cyclin–dependent kinases (cdks). Three primary checkpoints, G_1_/S, G_2_/M, and the Metaphase checkpoint, operate in succession to ensure DNA stability and cellular integrity is maintained throughout the cell cycle [25], [26]. If development under low pH environments is genotoxic, we can hypothesize that one of these checkpoints would be activated, resulting in the developmental delay previously reported for sea urchin embryos.

Although changes in transcript levels are not expected to occur during these early cleavage stages, translation of pre-loaded, maternal mRNA for important mitotic regulators such as mitotic cyclins could very well be altered. The family of mitotic cyclins (M-cyclins) is comprised of three distinct isoforms (A, B, B_1_) and was first identified in sea urchins as proteins that are rapidly synthesized and degraded at mitosis during the first cell cycle [27]. The concentration of M-cyclins elevate in late G_2_ and peak during M-phase of the cell cycle where they complex with cdk1 to promote the G_2_/M transition and progression through M-phase [28]. Loss of cyclin–B, in urchins and *Drosophila*, results in a developmental “lag” in which embryos proceeded more slowly through developmental programs when compared to wild type embryos [29], [30]. Given that the reduction in cleavage rates seen in urchin embryos raised under low pH conditions are reminiscent of this developmental lag, we can hypothesize that similar mechanisms are involved and would predict that cyclin-B levels differ significantly between embryos developing in control and low pH seawater.

Lastly, microtubule dynamics are highly sensitive to environmental perturbation and can play a role in the final stage of cellular arrest [31], [32]. The mitotic spindle (or Metaphase) checkpoint occurs at the point in mitosis where mitotic spindles should have formed correctly and positioning of condensed chromatin along the metaphase plate has occurred. The spindle checkpoint is one of the final cell cycle regulatory mechanisms prior to cytokinesis and failure of normal spindle formation can block transition of the cell cycle from metaphase to anaphase [33], [34]. Thus, if low seawater pH affects microtubule dynamics and results in the activation of the metaphase checkpoint, we would predict a disproportionate number of malformed mitotic spindles in embryos developing at a pH that correlates to the reduction in cleavage rates.

In order to test these hypotheses for how mitotic control mechanisms may be activated by low pH environments during early sea urchin development, we monitored cell cycle progression using these three cellular metrics in *S. purpuratus* single celled embryos. In doing so, we integrated common cellular and developmental methods previously established in sea urchin embryos to test the hypotheses described above and to determine if common cell cycle checkpoints are involved in the reduction of mitotic cleavage previously observed in echinoderm embryos.

## Results and Discussion

### Sea urchin development under low pH environments

**Figure 1:**
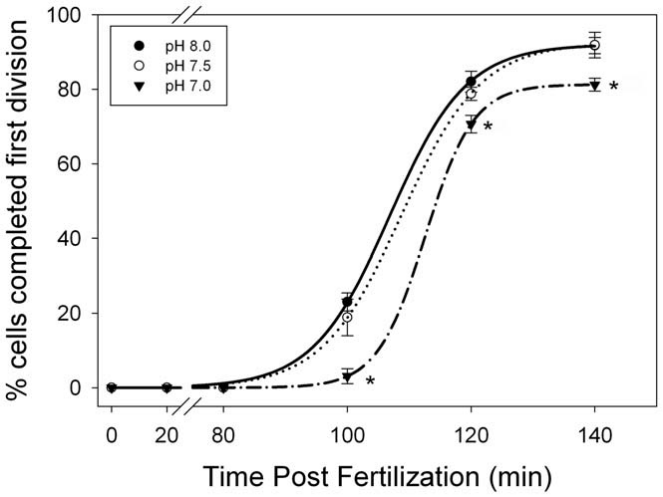
Progression of the first mitotic division in *S. purpuratus* embryos developing in seawater with differential pH. The number of sea urchin embryos completing the first mitotic cleavage was monitored for 140 min post fertilization. Immediately after fertilization replicate cultures from five independent female urchins were raised in seawater infused with CO_2_ and maintained at a control pH (8.0, solid circles), mid-level pH (7.5, open circles), or a low pH (7.0, solid triangles). At 10 min intervals, a minimum of 300 embryos from each culture were assessed to determine if they had completed the first cellular division. Kaplan-Meier log-rank survival analysis found a statistically significant difference between the timing of the first mitotic division between treatments (p<0.001). Post-hoc pairwise multiple comparison tests found a significant difference between the pH 7.0 treatment and both pH 7.5 and 8.0 treatments (p<0.00001, indicated by asterisks).

To verify similar reductions in the rate or number of cleavage events in *S. purpuratus* embryos reared in low pH seawater are observed in our system, we tracked the synchronous development of sea urchin embryos for 140 min post fertilization and determined the number of embryos that had successfully completed the first mitotic division at 10 min intervals. Similar to results previously reported for the sea urchin *Heliocidaris erythrogramma* [35], [36] no significant changes in either the rate of cleavage, or the total number of embryos successfully completing the first cellular division was observed between embryos reared in control seawater (∼pH 8.0) or in seawater maintained near a pH of 7.5 by CO_2_ infusion (Fig. 1). Consistent with previous reports, development of urchin embryos reared in low pH seawater was negatively impacted [37]–[40]. When reared at a pH of 7.0, *S. purpuratus* embryos displayed a statistically significant reduction in both the rate of cleavage (∼8 min), indicated by a shift in the curve to the right, and the total number of successful mitotic divisions (Fig. 1). For embryos cultured at a pH of 8.0 and 7.5, the percentage of cells that had completed the first division by 120 min was 82.039±1.613 and 78.77±1.002 respectively, while embryos cultured in seawater held at a pH of 7.0 showed a significant decrease, with only 70.634±1.36% completing the first division by 120 min. At 140 min, the gap between embryos developing at pH 8.0 and pH 7.0 remained statistically significant, with 91.72±1.24% (pH 8.0), 91.85±1.98% (pH 7.5) and 81.21±1.01% (pH 7.0) of embryos completing the first division. Kaplan-Meier log-rank survival analysis identified a significant difference between the timing of the first mitotic division between treatments, with the median time for division occurring within 121.045 min (±0.584) for embryos cultured at a pH of 8.0, 121.144 min (±0.325) for embryos cultured at pH 7.5, and 133.056 min (±0.226) for embryos cultured at pH 7.0. The log rank statistic for the survival curves is greater than would be expected by chance, thus there is a statistically significant difference between the timing of the first mitotic division among treatments (X^2^_2, 300_ = 31.637, p<0.001). Post-hoc analysis performed using a Bonferroni pairwise multiple comparison found no significant difference between pH 8.0 and 7.5 treatments (p = 0.863). However, there was a significant difference between the pH 7.0 treatment and both the pH 8.0 (p<0.00001) and pH 7.5 (p<0.00001) treatments. No significant effect of female was seen within a treatment (p = 0.920).

By fertilizing the eggs in seawater maintained at the control pH (8.0) prior to splitting the batches into CO_2_ treated seawater, we were able to eliminate known impacts on sperm motility as a confounding factor (1). These results are consistent with previous studies and confirm low pH seawater can slow the developing embryo within the first hour post-fertilization. Our data also suggest that the impact occurs via two mechanisms, reducing the developmental rate of cleavage events in addition to the total number of successful divisions when the pH falls below 7.5. Although we did not track the development long enough to ascertain if this was indicative of a reduction in survival of embryos at this early stage, comparative data on sea urchin species ranging from tropical to polar species suggests larval survival is unaffected at this pH range [4], [10], [38]. Thus, the embryos failing to divide may be entering an extended resting phase (G_0_) in which the cell has left the cell cycle and stopped dividing until more favorable conditions allow re-entry into the cell cycle. Alternatively, some developing embryos observed under the microscope showed characteristic morphological changes including blebbing and shrinkage that is consistent with the onset of apoptosis (data not shown). Thus, induction of apoptotic pathways in sea urchin embryos developing under low pH environments is an alternative fate, and further investigation would be necessary to determine whether low pH leads to cell death or stasis in zygotes that fail to divide in the normal time frame.

### Onset of DNA synthesis

**Figure 2:**
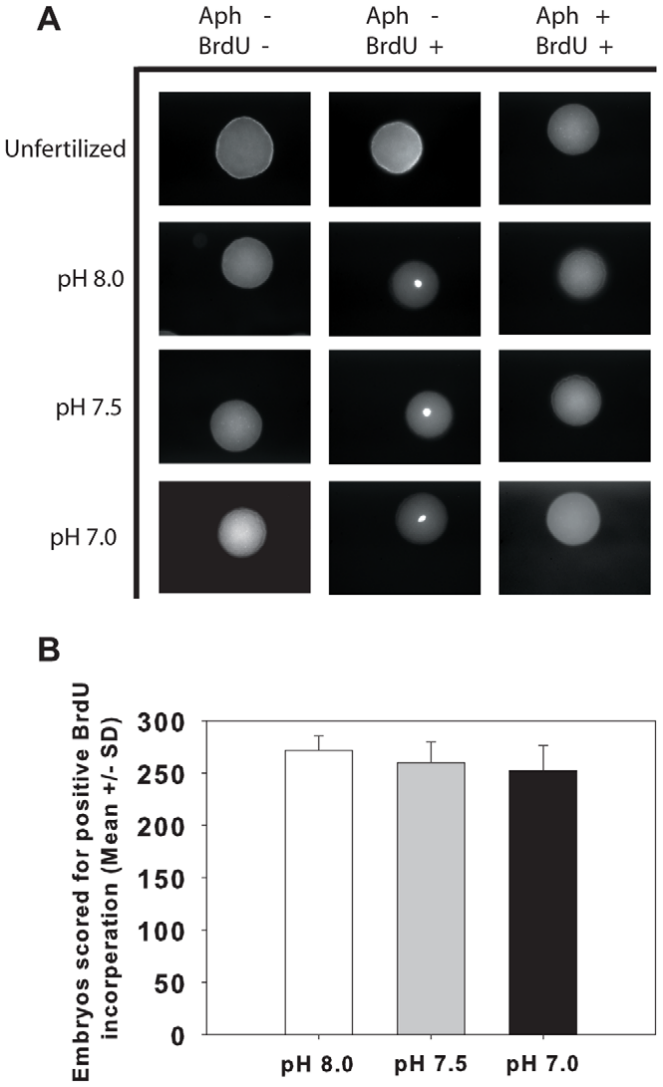
Impact of low pH on DNA synthesis. The onset of DNA synthesis in embryos developing in different pH seawater was monitored by incorporation of a modified uridine molecule, BrdU, during DNA replication. (A) No BrdU incorporation was detected in embryos developing in the absence of Brdu (BrdU−). Embryos developing in the presence of BrdU alone show strong incorporation into newly synthesized DNA within 30 minutes of fertilization, suggesting DNA synthesis is unaffected. When embryos were allowed to develop in the presence of both BrdU and the DNA synthesis inhibitor, Aphidocholin (BrdU+/Aph+), no BrdU incorporation was observed, suggesting the positive BrdU incorporation was due to synthesis of new DNA and not the activity of DNA repair enzymes. (B) Scoring of embryos (n = 300 per experimental culture) show no statistical difference in the number of BrdU positive embryos across treatments: pH 8.0 (white bars) pH 7.5 (grey bars) pH 7.0 (black bars).

To determine if the low pH of external seawater infused with high levels of CO_2_ could disrupt the intracellular rise in pH that is required for the onset of protein synthesis and ultimately DNA synthesis, we used the incorporation of 5-bromo-2′-deoxyuridine (BrdU), a synthetic nucleotide and thymidine analoge, into newly formed DNA strands to track the onset of DNA synthesis. No positive staining of the cell nucleus was observed in unfertilized embryos or negative control embryos (Fig. 2A). In embryos developing in the presence of BrdU, strong positive nuclear staining was seen in all pH treatments (Fig. 2A). For each replicate culture (n = 5 per treatment), 300 embryos were scored for positive BrdU incorporation 30 min post-fertilization. Counts were performed in triplicate by independent observers and no significant difference was observed across treatments (Fig. 2B, two-way ANOVA, *F*_2,14_ = 1.931, p = 0.207) or female (*F*_4,14_ = 1.025, p = 0.450). To ensure the BrdU positive cells were a result of newly synthesized DNA and not a false positive created by the incorporation of BrdU through the activity of DNA repair enzymes, we incubated a subset of embryos (negative controls) with BrdU and Aphidicolin, a chemical know to specifically block DNA synthesis while allowing DNA repair enzymes to function normally [41]. No BrdU positive nuclei were observed in the negative control embryos (Fig. 2A). These results suggest that pH levels as low as 7.0 do not activate the G_1_ restriction checkpoint or inhibit the G_1_/S transition during the initial stages of the cell cycle. As no significant differences were observed in the onset of DNA synthesis, it is unlikely low external pH environments compromise the internal pH of the developing embryo, which has been directly linked to the onset of both DNA and protein synthesis in *S. purpuratus* embryos [18], [19]. These data suggest an alteration in internal physiological functioning of the embryo has occurred to compensate for the potential internal acidosis brought on by the hypercapnic conditions. Sea urchins are relatively poor acid-base regulators [42]–[45] however, and alterations in acid-base status may actually be secondary effects brought on by physiological adjustments invoked for ionic and osmotic regulation [46].

One potential family of ionic and osmotic regulators that may play an important role in maintenance of intracellular pH includes ATPases such as Na^+^/K^+^-ATPase and H^+^/K^+^-ATPase transporters. These ion-regulatory enzymes couple the hydrolysis of ATP to the movement of ions against a gradient and therefore any up-regulation of their cellular activity could come at a significant energetic cost. For instance, the Na^+^/K^+^ transporter alone can account for as much as 40% of an organism's ATP consumption [47] and thus, long-term activation of these pathways could significantly affect the energy available for growth and proliferation, especially in non-feeding larval stages. While these mechanisms may provide immediate relief from hypercapnic environments, it is unlikely they would play a major role in maintaining the intracellular conditions of a developing embryo for extended periods. In fact, in an apparent effort to maintain energy reserves during cellular acidosis, some invertebrates actually shift from energy demanding ATPase transporters to more energy efficient ion exchangers such as Cl^−^/HCO_3_^−^ and Na^+^/H^+^, the antiporter responsible for the initial intracellular pH rise in fertilized eggs [48]. Similar molecular shifts have also been identified in sea urchin larvae raised under high CO_2_ environments. Todgham and Hofmann [9] report mRNA levels for a variety of ATPases display a marked decreased in expression in larvae of *S. purpuratus* by the time they have reached early prism, while expression of Na^+^/H^+^ and Cl^−^/HCO_3_^−^ transporters were unaffected. Thus, a primary mechanism of acid-base regulation in marine invertebrates may rely on secondary active transport by ionic regulators such as Na^+^/H^+^ and Cl^−^/HCO_3_^−^ antiporters to compensate for the lowering of intracellular pH as a result of elevated pCO_2_. Support for this mechanism can be found in crustaceans that utilize similar anion exchange processes to restore haemolymph pH to normoxic conditions after acid-base imbalances [49], [50]. Given our previous understanding of the role these ion transporters play in cellular homeostasis, further experiments that integrate internal measurements of cellular pH with targeted inhibition of transporters and antiporters will play an important role in our understanding of the capacity of developing marine larvae to mitigate environmental changes in pH.

### Temporal activity of the mitotic regulator cyclin-B

**Figure 3:**
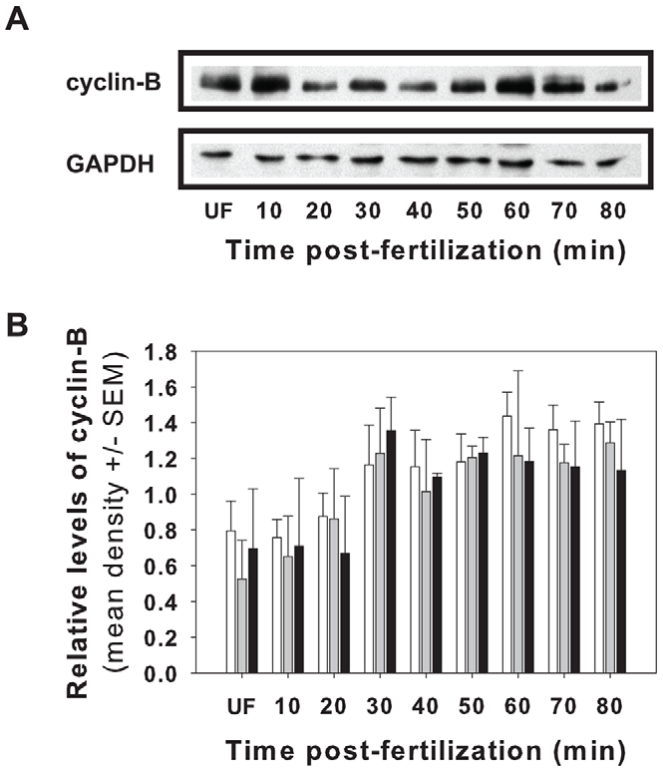
Relative level of cyclin-B protein expression. The temporal changes of the mitotic regulator cyclin-B was determined during the first cellular cleavage of urchin embryos developing in seawater maintained at various pH levels. (A) No difference in the expression of cyclin-B protein relative to GADPH was observed between embryos developing at a pH of 8.0 (white bars), 7.5 (grey bars) or 7.0 (black bars). (B) A representative Western blot showing the specificity of the primary antibodies.

Cyclin/Cdk protein complexes operate to ensure earlier mitotic events are properly completed and that cellular integrity is maintained before initiation of subsequent events. Activation of these checkpoints can result in transient delays in order to provide extended periods for damage to be repaired prior to progression to the next phase of the cell cycle. To test the effects of development under high CO_2_ conditions on the activities of cyclin-B/cdk1 complexes that control the G_2_/M transition, protein extracts were prepared from control embryos (pH 8.0) and embryos reared at a pH∼7.5 or 7.0. Relative changes in the level of cyclin-B protein were then measured by Western blot analysis using an affinity-purified anti-cyclin-B_1_ polyclonal antibody along with an anti-GAPDH antibody to control for variation in protein loading. In all treatments, a single protein of ∼46 kDa was detected by the cyclin-B_1_ antibody (Fig. 3A). Immunoblots of embryos removed at regular intervals during the first 90 minutes of development show temporal changes in cyclin-B levels consistent with previous findings [29], [30]. Cyclin-B protein is detectable even in unfertilized embryos, consistent with pre-loading of maternal RNA and protein (Fig. 3A). Within 1-hour post-fertilization, the level of cyclin-B protein relative to GAPDH increases by ∼15% in embryos independent of the seawater pH (Fig. 3B). Comparison of the relative level of cyclin-B between treatments for each time-point showed no significant differences as a function of seawater pH (two-way ANOVA, *F*_2,14_ = 0.156, p = 0.859) or female (*F*_4,14_ = 1.944, p = 0.223) and suggest the temporal activation of M-cyclins is not impacted by low pH environments.

The three distinct gene families that comprise mitotic cyclins share some functional overlap [51]. While the functional overlap of these proteins may be able to compensate for the loss of another, there may be a developmental cost associated with this compensation. Complete cell cycle progression has been observed in sea urchins and *Drosophila* despite the loss of cyclin-B, presumably due to the compensation of cyclin-A [29], [30]. However, in each case a developmental “lag” was observed in which embryos proceeded more slowly through developmental programs when compared to wild type embryos [29], [30].

It is possible the reduction in embryo cleavage rates identified in this study is the result of changes in cyclin-A levels and not cyclin-B. Voronina and colleagues [29] report similar and sometimes more pronounced effects on cell cycle progression in loss-of-function experiments involving cyclin-A. This is unlikely to be the case here however, given the role of cyclin-A during early S-phase regulation of DNA synthesis in addition to the G_2_/M transition. Cyclin-A is known to associate with two different cdks (Cdk1 and Cdk2) and protein levels steadily increase from S phase through late G_2_ [52], [53]. During the G_1_/S transition, cyclin-A is thought to activate Cdk2, which is essential for DNA synthesis [54], [55]. If cyclin-A depletion were playing a role in the reduction of cleavage rates reported here, we would expect to see significant delays in the onset of DNA synthesis. Furthermore, Cdk2 activation plays a fundamental role in the regulation of centrosome duplication. Thus if cyclin-A were implicated in these delays, we would expect also to see a reduction of mitotic spindle formation, which did not occur (see below).

### Mitotic spindle formation

**Figure 4:**
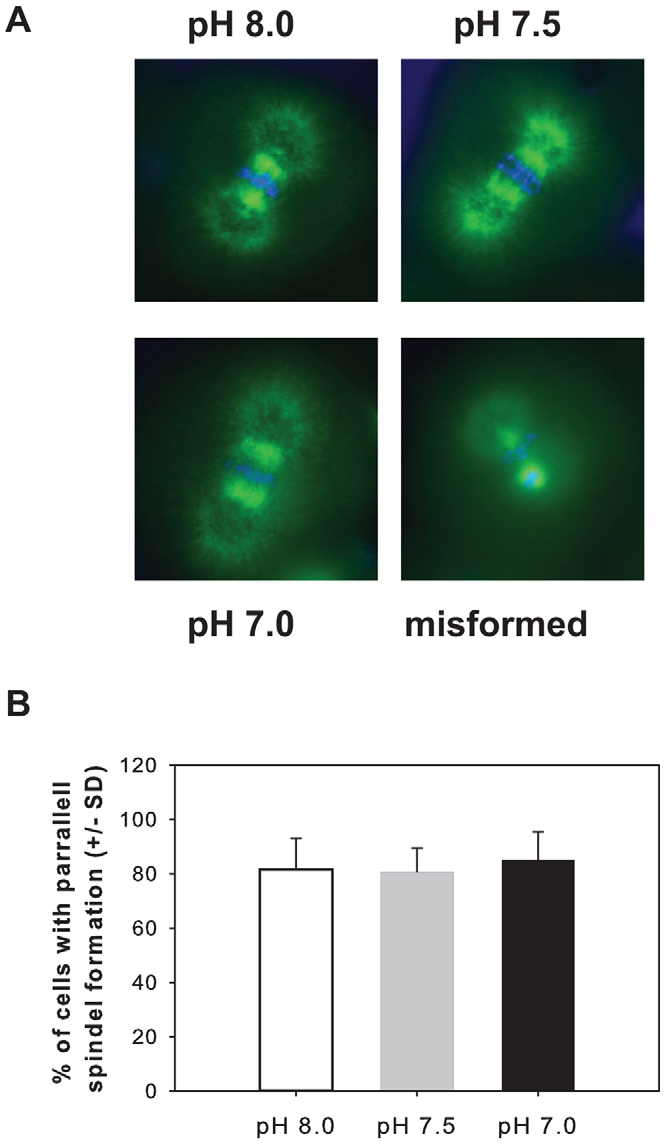
Impact of low pH on mitotic spindle formation. (A) Mitotic spindle formation was observed by staining the microtubules of sea urchin embryos during the first mitotic division. (B) Triplicate blind counts of a minimum 300 embryos from each culture found no significant difference in the number of malformed mitotic spindles observed in embryos reared in seawater held at a pH of 8.0 (white bar) 7.5 (grey bar) or 7.0 (black bar) for 90 min post-fertilization.

To characterize the effect a low pH environment has on microtubule dynamics, we also assessed mitotic spindle formation via immunohistochemical staining in developing embryos at 90 min post-fertilization. Triplicate blind counts of a minimum of 300 stained cells were independently scored for uniform polarity of microtubules in the spindle, with ends at or near the poles and extending toward the cell cortex or chromosomes (Fig. 4A). Normal alignment of condensed chromatin with respect to the mitotic spindle was also visualized by Hoescht staining (Fig. 4A). No significant differences in the number of embryos displaying normal mitotic spindle formation were observed for any of the pH treatments (two-way ANOVA F_2,14_ = 0.167, p = 0.783) or a particular female within a treatment (F_4,14_, = 1.650, p = 0.408). On average, 81.63±6.61% of embryos developing in pH 8.0 seawater displayed normal microtubule staining, while 80.73±5.02% and 85.13±5.91% of embryos were scored as normal in pH 7.5 seawater and pH 7.0 seawater respectively (Fig. 4B).

Centrosomes, and microtubules in general, play an important role in late G_2_ checkpoints [32], [56]–[58]. Sudden disassembly of microtubules by chemical treatment is known to delay cell cycle progression for up to 4 h [59]. In our assessment of mitotic spindle formation, we found no indication that the nucleation of microtubules was inhibited at any pH. In the majority of embryos, spindle microtubules appear stable and normally oriented with complete extension towards the metaphase plate and it is not likely the spindle checkpoint is activated.

Cyclin-B also plays one additional role in the onset of cytokinesis that may still activate a checkpoint in late metaphase. Transition from metaphase to anaphase and the subsequent separation of sister chromatids is regulated by the spindle checkpoint primarily through the proteolytic activity of a specialized proteasome known as the anaphase-promoting complex (APC) [60]–[63]. Activation of the APC results in the ubiquitination and degradation of cyclin-B and subsequent inactivation of maturation promotion factor among other proteins involved in sister chromatid cohesion. Failure of the APC to reduce cyclin-B levels will block the exit of the embryo from mitosis [64], [65].

As we only followed the levels of cyclin-B protein for the first 90 min post-fertilization, we are unable to verify that a significant reduction in mitotic cyclins has occurred in our embryos. As a result, we cannot yet definitively exclude activation of the spindle checkpoint pathway via the APC. Furthermore, cyclin-B-Cdk1 activity is a major integration point for a number of upstream stress signaling cascades with cell cycle regulation pathways, such as the stress-activated MAP kinase pathways with modulators of G_2_ progression and transition [66]. As such, a number of alternative mechanisms will need to be extensively explored to definitively rule out a role for M-cyclins in transducing the effects of low environmental pH to the developing embryo.

### Conclusions

As seen with previous investigations, early development was only affected by a significant reduction in seawater pH<7.5 [4], [37]–[40]. Our results, along with those of preceding studies looking at later developmental stages, suggest early developmental stages of sea urchin larvae are robust with respect to the predicted changes in ocean pH by the year 2100. Near future reductions in sea surface pH by itself is therefore unlikely to impact embryonic development in sea urchin populations. It is still unclear, however, if reductions in ocean pH may act synergistically with changes of other climatic variables to exacerbate organismal stress, and so it remains germane to identify the mechanism by which low pH environments act to restrict developmental rates in some marine species. Common cell cycle checkpoints do not appear to play a role in the reduction of embryonic developmental rates in our study, and thus it is plausible that direct impacts on metabolism or energetic limitations to physiological responses are working to restrict development. Our data provide further support for the idea that energetic constraints shape the response and ultimately the success of marine organisms in a changing ocean.

Lastly, given the paucity of information regarding the physiological costs of synergistic stresses, it is premature to conclude these species will be immune to the expected near future ocean changes. These data serve to highlight the need to continue to integrate mechanistic studies of multi-stressor impacts to identify relevant physiological impediments to adaptation in changing environments.

## Materials and Methods

### Animals and Embryos

#### Ethics statement

All necessary permits were obtained from the state of California Department of Fish and Game prior to animal collection. Adult *S. purpuratus* were collected from Bodega Bay and shipped in chilled seawater to the University of South Carolina, where they were maintained in recirculating seawater tanks at 12°C. For these experiments, eggs from five separate females were fertilized with sperm from a single male urchin and reared independently. Methods for procurement and culturing of echinoderm eggs were followed as described by Foltz and colleagues [67]. Spawning was induced by injection of 0.55 M KCl into the coelomic cavity. Male sperm was collected dry, and kept on ice until used. Female eggs were collected by inversion over a beaker, gravity settled, washed three times in filtered seawater (FSW), and resuspended in FSW as a 10% v/v suspension. The egg suspension was de-jellied by passing through 125 µm nitex mesh 8 times, allowing eggs to gravity settle and decanting the supernatant. Egg suspensions were washed three times in FSW and resuspended in 10 volumes in artificial seawater (ASW) supplemented with 1 mM ATAZ to prevent hardening of fertilization membranes. A 1∶2,500 dilution of sperm was made with 10 ml of jelly water and 10 mL of ASW supplemented with 2 mM ATAZ then added to 20 mL egg suspension for a final sperm dilution ratio of 1∶5,000. Only batches in which 95% of embryos displayed a fertilization envelope within the first 2 min post-fertilization were used to ensure synchronous development. Fertilization envelopes were removed by passing freshly fertilized eggs through 210 µm nitex mesh 20 times. Next, embryos were immediately separated into 3 equal volumes; hand centrifuged to remove the supernatant and resuspended as a 1% suspension in 250 mL of CO_2_ treated FSW at an approximate pH of 8.0, 7.5, or 7.0, capped and allowed to develop in an environmental chamber at 15°C. In total, the experiment consisted of 15 experimental cultures, 5 replicate cultures, one from each female, for each of three pH treatments. Although these experiments were designed within an ecological framework, it is common practice to remove the jelly coat, which naturally diffuses in seawater over time, and the fertilization envelopes to perform sensitive immunohistochemical staining protocols. Failure to remove these external structures can significantly interfere with uptake of antibodies and increase background fluorescence. Given the major contribution of these components occurs during fertilization (activation of the acrosome reaction in sperm) and polyspermy block [68], [69], it is unlikely removal of the components would lead to an impact on cellular homeostasis in relation to pH.

### Seawater Chemistry

To parameterize the seawater conditions experienced by the developing embryos during the experiment, temperature, pH_T_ (total scale), salinity and total alkalinity (TA) were measured for each culture immediately after removal of the final aliquot of embryos. The water chemistry parameters for each culture are reported in Table 1. Temperature was measured with a calibrated thermocouple (Omega HH82A). Mean temperature across all cultures was 15.1±0.8°C–15.15±0.17°C. Salinity, measured with a benchtop salinity meter (YSI 3100), was 34.17±0.23 ppm. For *p*CO_2_ analysis, we followed Standard Operating Procedures (SOPs) for pH (SOP 6b) and TA (SOP 3b) [70] as modified in Fangue et al. [71]. CO2calc [72], using the constants of Mehrbach et al. [73] as refit by Dickson and Millero [74], was used to calculate all other carbonate parameters. Filtered seawater (0.22 micron) used in these experiments was originally obtained from the Belle W. Baruch Institute for Marine & Coastal Sciences, which sits on the North-Inlet of Winyah Bay. Artificial seawater used during fertilization and removal of fertilization envelopes of embryos was generated as previously described by Foltz and colleagues [67]. Combination of these two sources of seawater during egg washing and setting of final cultures may have lead to some of the variation in seawater parameters within treatments (Table 1).

**Table 1:** Seawater chemistry for larval experiments.

	Salinity (ppm)	Temp (°C)	pH	TA	pCO_2_ (µatm)
**Control pH**					
female 1	34.7	15.4	8.07	2170.08	346.73
female 2	34.2	15.0	8.04	2118.59	378.41
female 3	34.1	14.9	8.02	2004.66	367.58
female 4	34.0	15.1	7.99	2038.32	399.98
female 5	34.2	15.2	8.01	2122.71	397.63
**Mean±SD**	**34.24±0.03**	**15.14±0.19**	**8.03±0.03**	**2090.87±67.55**	**378.07±22.11**
**Mid-level pH**					
female 1	34.2	15.4	7.47	2107.81	1287.14
female 2	33.8	15.1	7.45	2060.10	1564.76
female 3	34.1	14.9	7.52	2070.50	1792.70
female 4	34.0	15.2	7.4	2050.30	1373.94
female 5	34.2	15.1	7.51	2103.88	1414.79
**Mean±SD**	**34.06±0.05**	**15.20±0.18**	**7.47±0.05**	**2078.52±25.98**	**1486.66±198.39**
**Low pH**					
female 1	34.6	15.4	7.1	2025.64	4254.64
female 2	34.2	15.0	7.09	2053.77	3694.12
female 3	33.9	15.3	7.02	2063.68	4438.79
female 4	34.1	15.2	7.04	1922.53	3924.47
female 5	34.3	15.1	7.04	2084.06	4239.20
**Mean±SD**	**34.22±0.03**	**15.12±0.16**	**7.06±0.03**	**2029.93±63.62**	**4110.24±297.11**

Carbonate parameters for replicate larval cultures reared under each seawater pH treatment are shown. The final row reflects the Mean values ± SD for each culture within a treatment (n = 5 females). Total alkalinity (TA), pH, temperature, and salinity were measured parameters and the remaining parameters were calculated using CO2calc.

### BrdU incorporation

To determine if low pH has an impact on DNA synthesis, we utilized a modified protocol from Zhang et al. [41] to incorporate 5-bromo-2′-deoxyuridine (BrdU, Invitrogen) into newly replicated DNA. Immediately after setting the final embryo cultures as described above, three, 3 ml aliquots were removed from each treatment for all five females and transferred to a glass culture tube and allowed to develop at 15°C in the presence or absence of 0.1 mg/ml BrdU for 40 min. To control for false positives resulting from the incorporation of BrdU into DNA as a result of DNA repair activity, DNA replication was blocked by the addition of Aphidicolin (Sigma, final concentration 20 µg/ml) to the remaining aliquot of developing embryos. At 10 min intervals, 50 µl aliquots were removed from the culture tubes and fixed in 4N HCl for 2 h at room temperature. Following fixing, the HCl was removed and embryos were covered with 100% methanol and incubated at −20°C overnight. Next, eggs were washed 5 times for 5 min each in phosphate buffered saline containing 1% bovine serum albumin (PBS-1% BSA), and then blocked in PBS-1% BSA for 1 h at RT. The block was removed and the embryos were incubated in primary antibody (anti-BrdU mouse IgG-alexa fluor 546 conjugate, Invitrogen) diluted to a final ratio of 1∶133 in PBS-1% BSA supplemented with Donkey Serum (1∶60) for 1 h at RT. Eggs were then washed 12×5 min in 1% PBS-Tween 20. Approximately 10 µl of eggs were diluted in 20 µl of a 1∶1 Glycerol:PBS solution and mounted on acid washed slides which were sealed and maintained in the dark at 4°C prior to fluorescent microscopy visualization. Control embryos (pH 8.0) were initially visualized to identify the time point with the greatest proportion of cells with positive staining of nuclear DNA. For these experiments, the 30 min time point was used to score embryos for all treatments.

### Western analysis

At 10 min intervals, 1 ml aliquots of suspended embryos were removed from the cultures described above, centrifuged at 12,000 g for 1 min, seawater removed and then flash frozen in liquid nitrogen. All samples were stored at −80°C until used for protein extraction. Total cellular protein was extracted by homogenization of frozen embryos in 5 volumes RIPA buffer [150 mM NaCl, 50 mM Tris (pH 8.0), 1.0% IGEPAL, 0.5% deoxycholate, 0.1% SDS] by passage of embryos several times through an 18 gauge and then a 22 gauge needle. Homogenates were centrifuged at 12,000×g for 10 min and supernatant was transferred to a new tube. Protein concentration was determined by Bradford assay using Commasie Plus protein assay reagent (Thermo Scientific). Twenty-five µg of protein was separated by SDS-PAGE and transferred to PVDF membrane (GE Healthcare). Immunochemistry was performed using a SNAP i.d. protein detection system (Millipor) following manufacture's recommendations using the following antibodies: (1°) anti-cyclin-B1 (Santa Cruz Biotechnology, sc-595) and (2°) goat anti-rabbit IgG-HRP (Santa Cruz Biotechnology, sc-2004). Protein detection was performed using SuperSignal West Pico chemiluminescent substrate (Thermo Scientific) and exposure to clear blue X-Ray film (Thermo Scientific). Densitometry was performed on a Fotodyne Investigator imager with accompanying software. The membranes were then stripped by incubation in 0.2 M NaOH for 5 min with gentle agitation and re-probed with anti-GADPH (1°) (Santa Cruz Biotechnology, sc-48167) and goat anti-rabbit IgG-HRP (2°) (sc-2004) to correct for loading variation. Relative levels of cyclin-B adjusted for loading variations are reported as ratio of cyclin-B to GADPH.

### Tubulin staining

To assess the potential for reduced seawater pH to impact the normal formation of mitotic spindles, 1 ml aliquots were removed in 10 min intervals starting at 60 minutes post-fertilization. Embryos were then immediately fixed in 3.2% formaldehyde made fresh prior to use and stored at 4°C overnight. Cells were then washed 3 times in PBST (phosphate buffered saline, 0.1% Triton-x-100) and stored at 4°C until stained. Prior to staining cells were treated with 0.1% NaBH4 in PBST for 4 h with steady agitation to reduce auto-fluorescence. Cells were washed 3 times for 20 min in PBST then blocked in PBST supplemented with 5% goat serum overnight at 4°C. Cells were incubated in monoclonal mouse anti-α-tubulin antibody (clone B-5-1-2, Sigma) in PBST at a 1∶5000 dilution for 24 h with gentle agitation, followed by two 5 min washes and one overnight wash at 4°C in PBST. Embryos were next incubated with the secondary antibody (Fluorescein-conjugated goat anti-mouse IgG, Cappel #55514) for 24 h followed by two 5 min washes and one overnight wash at 4°C in PBST in the dark. Chromatin was also stained by 10 min incubation in 0.01 mg/mL Hoescht dye followed by 3 quick washes in PBST. Cells were then transferred to slides and mounted with Invitrogen Prolong Gold anti-fade reagent and incubated at 37°C for 4 h. Mounted, stained cells were stored at room temperature in the dark until visualized by fluorescent microscopy. Control embryos (pH 8.0) were initially visualized to identify the time point with the greatest proportion of cells with positive staining of microtubule formation. For these experiments, the 90 min time point was used to score embryos for all treatments. High-resolution images of a minimum of 300 individual cells were captured using ImageJ software. Cells were scored for normal mitotic spindle formation by triplicate blind counts.
